# Dynamic Flow Characteristics and Design Principles of Laminar Flow Microbial Fuel Cells

**DOI:** 10.3390/mi9100479

**Published:** 2018-09-20

**Authors:** Way Lee Cheng, Celal Erbay, Reza Sadr, Arum Han

**Affiliations:** 1 Department of Mechanical Engineering, Texas A&M University at Qatar, Doha, Qatar; cheng-wl@alum.northwestern.edu; 2 TUBITAK-Informatics and Information Security Research Center, Kocaeli 41470, Turkey; celal.erbay@tubitak.gov.tr; 3 Department of Mechanical Engineering, Texas A&M University, College Station, TX 77843, USA; 4 Department of Electrical and Computer Engineering, Texas A&M University, College Station, TX 77843, USA; arum.han@ece.tamu.edu

**Keywords:** laminar flow microbial fuel cell, computational fluid dynamics, microfluidics

## Abstract

Laminar flow microbial fuel cells (MFCs) are used to understand the role of microorganisms, and their interactions with electrodes in microbial bioelectrochemical systems. In this study, we reported the flow characteristics of laminar flow in a typical MFC configuration in a non-dimensional form, which can serve as a guideline in the design of such microfluidic systems. Computational fluid dynamics simulations were performed to examine the effects of channel geometries, surface characteristics, and fluid velocity on the mixing dynamics in microchannels with a rectangular cross-section. The results showed that decreasing the fluid velocity enhances mixing but changing the angle between the inlet channels, only had strong effects when the angle was larger than 135°. Furthermore, different mixing behaviors were observed depending on the angle of the channels, when the microchannel aspect ratio was reduced. Asymmetric growth of microbial biofilm on the anode side skewed the mixing zone and wall roughness due to the bacterial attachment, which accelerated the mixing process and reduced the efficiency of the laminar flow MFC. Finally, the magnitude of mass diffusivity had a substantial effect on mixing behavior. The results shown here provided both design guidelines, as well as better understandings of the MFCs due to microbial growth.

## 1. Introduction

Microbial bioelectrochemical systems (BESs) utilize electrochemically active microorganisms to extract energy harbored in various carbon sources, especially in wastewater. Electrons generated during the natural metabolic processes of these microorganisms can be extracted in the form of electricity when the system is configured as a microbial fuel cell (MFC) [1,2], hydrogen when configured as a microbial electrolysis cell (MEC) [3], or even as a driving force for saltwater desalination when configured as a microbial desalination cell (MDC) [4]. In all of these systems, the biofilm is formed on the anode surface through microbial attachment and growth, which plays a crucial role in BES performances and operations. However, how various operating parameters, including flow conditions, can affect this biofilm over time, and how this biofilm influences the overall system performance, is still not very well understood [5,6,7]. The limited understanding of the effects of the growing biofilm is partially due to the limited availability of experimental systems, which can be used to study such relationships.

Laminar flow MFCs fall within the category of microscale fuel cells, which are being developed as potential portable energy sources or small-sized power sources capable of continuous operation for long periods of time [8,9,10,11,12]. Whether laminar flow MFCs can truly be used as energy harvesting devices is significantly debated. However, more importantly, laminar flow MFCs are ideally suited as experimental tools for studying microbial biofilm in BESs. With the top cover of typical laminar flow MFCs being transparent, microbial attachment and biofilm growth on anodes can be directly monitored in these systems, whilst simultaneously measuring the system performances under various conditions [9]. The electrical current generated in the MFC is directly proportional to the hydrogen produced in an MEC, or the degree of desalination in MDC. Therefore, the results obtained from the study of laminar flow MFCs can be directly utilized to estimate the performance of most BESs, beyond just MFCs. Thus, laminar flow MFC can be a powerful tool to conduct an in-depth analysis of certain parameters directly affecting BES performances, such as biofilm (growth, biological, and/or biochemical activities) or other geometrical and fluid flow parameters [9,10].

The main requirement of a laminar flow MFC is that the two electrolytes (anolytes and catholytes) within the system flow in a laminar flow state, with the anode and cathode positioned on each side of the diffusion boundary between the laminar flow. For example, wastewater flows on the anode side where microbes are attached to the electrode, whilst catholytes, such as ferricyanide which function as electron acceptors, flow on the cathode side [8]. The laminar flow characteristics of the microchannel flow allow the anolyte and catholyte streams, having different properties, to flow in parallel with a pure liquid–liquid diffusive interface, without the need for a proton exchange membrane (PEM), which typically separates anolyte from catholyte in conventional two-chamber MFCs [13]. This diffusive interface broadens in the stream-wise direction, and scales as the one-half power in the core region, and as the one-third power in the near-wall region [14]. This laminar flow interface eliminates the need for a PEM, hence the efficiency of such a system can be significantly higher than the conventional two-chamber MFC, as PEMs are the major source of low efficiency in MFCs. However, lower fluid velocity results in a wider inter-diffusion zone as the liquids are in contact with each other for a longer time, which causes the broadening of the mixing zone in the near-wall region [15]. Therefore, a reasonably high flow velocity is required for the liquid–liquid interface to function properly as a barrier, allowing only proton exchange, whilst minimizing mixing between anolyte and catholyte. The fluid flow in a typical laminar flow device is a diffusion-limited process, where a longer length is needed to achieve complete mixing [15]. Small Reynolds number and large Péclet number are the major characteristics of fluid flows in a typical microchannel system. Small Reynolds number means laminar flow with strong viscous effects and negligible inertia effects, and a large Péclet number implies high streamwise convective velocity and weak traverse diffusion rate.

Computational fluid dynamic (CFD) methods have been used in the past to analyze laminar flow fuel cells. Most of the literature analyzing the three-component microchannel system is focused on solving the continuity, two-dimensional Navier-Stokes, and the advection–convection equations [16,17]. CFD was also used to develop models for optimizing fuel utilization with minimal mixing between the fuel/oxidant streams, for various microchannel configurations [18]. Results of these simulations suggest that it is possible to improve the performance of a micro-fuel cell for rectangular cross-section channels, for cases of the high ratio of channel width to channel height, as well as higher Péclet number [19]. Kjeang et al. [16] presented a computational study of microfluidic biofuel cells using a two-dimensional finite element model, where the outcomes of the study provided a guideline for future biofuel cell design. However, in the literature, all MFC studies, both experimental and numerical, have a major limitation: They are conducted for a select few geometries or fluid flow cases. Therefore, the conclusions presented in those studies are only valid for those particular cases and may not be extended to dimensions or conditions not specifically covered in those studies.

More importantly, microfluidic MFCs pose unique challenges, which are non-existent in conventional microfluidic fuel cells, namely the growth of microbes and biofilm on anodes over time. The increase in biofilm thickness over time on the anode side creates an asymmetric flow profile, which poses a challenge to properly designing laminar flow MFCs and their operating conditions. An added challenge is that since biofilm grows over time, the flow behaviors near the biofilm, and thus the overall system performances, can also change over time. In addition, microbes are influenced, both negatively and positively, by shear stress induced by the flow, as summarized in Table 1. In a typical operation, higher flow speed can result in a longer and narrower diffusion boundary, and thus is desirable as they provide uniform experimental conditions throughout the length of the system. However, the higher flow speed is also directly proportional to higher shear stress within a confined channel that is widely known to, both positively and negatively, influence biofilm growth, detachment, and physiology [10,20,21,22,23,24,25].

In this study, we present a CFD-driven laminar flow MFC model in the presence of growing biofilm, which can be used as a general design guideline for these systems, and for the design of experiments to study how biofilm influences BES system performances. This work presents overall characteristics of the mixing layer in a multi-channel continuous-flow BES system, under different flow conditions and channel geometries. The effects of fluid velocity, fluid properties, channel configuration, and biofilm growth on the flow mixing zone, as well as on the shear stress on the biofilm, are investigated. All results are presented in terms of proper normalized parameters for the fluid flow and channel geometry conditions, to provide a more general view on the effects of channel geometry and flow conditions on the flow field. This will provide, for the first time, a comprehensive design guideline for the development and operation of microfluidic BES systems, and provide a unique perspective into how fluid flow is influencing, and is influenced, by the dynamically changing environment, such as biofilm growth over time.

## 2. Materials and Methods

### 2.1. Laminar Flow Microfluidic Channel System

**Figure 1:**
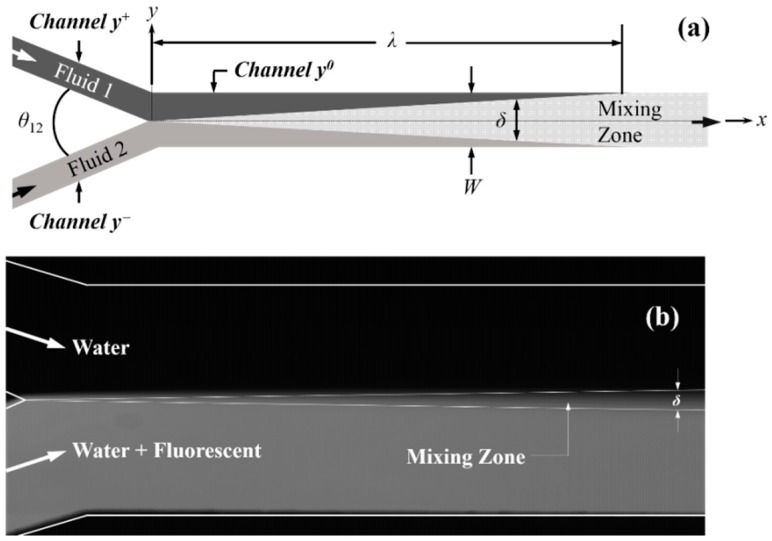
(**a**) An illustration of the dual-inlet laminar flow fuel cell system with parameters defined. (**b**) Experimental image showing the mixing zone between two parallel flow water streams, one of which is mixed with fluorescent.

Figure 1a shows a laminar flow microfluidic channel system consisting of three rectangular channels: Fluid 1 and 2 flows through inlet *Channels y*^+^ and *y*^−^ joining at *x* = 0 with an angle *θ*_12_ between the two channels, into *Channel y*^0^. Both, *Channels y*^+^ and *y*^−^ have a similar cross-section of *H* × *W*/2, whilst the cross-section of *Channel y*^0^ is *W* × *H*. Two fluids (anolyte and catholyte) entered *Channel y*^0^ via the inlet channels *y*^+^ and *y*^−^, respectively, and were mixed there within the mixing zone. Two commonly used channel geometries are often called Y- and T-shaped systems, where *θ*_12_ = 60° (or similar) and *θ*_12_ = 180°, respectively. Diffusion between the two fluids occurs after they enter *Channel y*^0^, with a mixing zone width of *δ*, which gradually grows, whilst the diffusion progresses in the channel. The length of the non-mixing zone, *λ,* is defined as the length of *Channel y*^0^, where a non-mixed zone exists within the channel. In this work, *λ* was chosen at the wall location, where less than 1% of fluid 1 and 2 were mixed. The mixing zone width, *δ*, was the distance between the boundaries of *C*_1_ = 0.99 and *C*_2_ = 0.99 (mass fractions for fluid 1 and 2, respectively) in the channel, at any downstream location of *x* < *λ*. Figure 1b shows an experimental image of the mixing zone between parallel streams of pure water and diluted fluorescent dye solution in a Y-shape microchannel, as shown in Figure 1a, where the diffusion thickness is labeled.

For applications in laminar flow MFC systems, only the portion of the *Channel y*^0^ where the two streams of fluids were unmixed can be used for power generation. It is therefore essential to investigate the effects of channel geometry, operational conditions, and biofilm characteristics in this region, on the flow characteristics.

### 2.2. Theoretical Background and Governing Equations

The governing equations for fluid flow in a laminar flow fuel cell system, assuming incompressible, steady-state, non-reacting, and constant properties, is given by the simplified Navier-Stokes equations,

∇·*u* = 0, (1)

*u*·∇*u* = − ∇*p* + *μ* ∇^2^*u*. (2)

where *p* is the pressure, *u* is the velocity vector, and *μ* is the dynamic viscosity. Species transport in *Channel y*^0^ is given by,

∇·(*ρ u C_j_*) = − ∇·*J_j_*, (3)

where *C_j_* is the local mass fraction of species *j* in the mixture, *ρ* is the fluid density, and *J_j_* is the diffusive flux of species *j* into the mixture. The Fick’s Law gives the diffusive flux, and invoking the dilute solution assumption, the diffusive flux is given by,

*J_j_* = − *ρD_j_* ∇*C_j_*, (4)

where *D_j_* is the diffusion coefficient of species *j* into the mixture. In general, an analytical solution is not available for the set of Equations (1) to (4), and thus is typically solved using numerical methods.

The most important parameter affecting hydrodynamics of the flow is the Reynolds number, a dimensionless velocity, representing the ratio of momentum and viscous forces, defined as

(5) $$Re=\;\frac{u\;D_{Hyd}}{\nu },$$

where *u* is the averaged fluid velocity and *D_Hyd_* is the hydraulic diameter of the channel defined as,

(6) $$D_{Hyd}=\;\frac{4\;A}{P}.$$

In Equation (6), *A* and *P* are the channel cross-sectional area and perimeter, respectively. Another parameter of interest, specifically for laminar flow MFC systems, is the surface shear stress, represented non-dimensionally as the friction coefficient, *C_f_*, defined as,

(7) $$C_{f}=\;\frac{2\;\tau }{\rho \;u^{2}},$$

where *u* is the characteristic velocity. In the current study, it might be more convenient to normalize the wall shear stress as:

(8) $$\tau^{*}=\;\frac{2\;W\;\tau }{\rho \;D_{j}\;u}.$$

The mixing of fluids 1 and 2 in *Channel y*^0^ is mainly characterized by two parameters, the length of the non-mixing zone, *λ,* and the width of mixing zone, *δ*, both shown in Figure 1. Due to the low Reynolds number for flows in these microchannels (typically *Re* < 2000), the mixing process was diffusion limited, and the parameter of interest was the Schmidt number, defined as,

(9) $$Sc=\;\frac{\nu }{D_{j}}.$$

The Schmidt number is the ratio of momentum and mass diffusivities, in the order of 100 to 1000 for typical liquids.

Table 2 lists the dimensions of the channels used in this work, as shown in Figure 1, unless stated otherwise. The hydraulic diameter of *Channel y*^0^, with the given dimensions, equals to 0.18 mm. Both fluids 1 and 2 were taken as pure water with a viscosity of 8.5 × 10^−7^ m^2^/s, and a having self-diffusion coefficient of 2.6 × 10^−9^ m^2^/s [26]. Note that if other fluids were used in place of water, the diffusion coefficient would be different and that changes the dimensional mixing characteristics of the two fluid streams. The width of *Channel y*^0^ was twice the width of both *Channel y*^+^ and *Channel y*^−^, and the Reynolds number of the flow was assumed to be 2, unless otherwise stated. Moreover, equal fluid velocities in both *Channel y*^+^ and *Channel y*^−^ were assumed. All simulation results are presented in normalized forms, covering a wide range of normalized input parameters (e.g., channel dimensions, fluid properties), so that it can be utilized in a broad range of cases.

The numerical solver in the commercial CFD package FLUENT was used to solve the set of differential Equations (1) to (4). The laminar flow and multi-component species transport models were used to model fluid flow within the channel system with smooth walls. Fluid properties of the mixtures were approximated with the mass (or volume) weighted mixing law. The binary diffusion coefficient was modeled using a constant multicomponent diffusion coefficient of 2.6 × 10^−9^ m^2^/s for water–water diffusion. The PISO scheme and second-order implicit scheme was applied for the pressure–velocity coupling and transient discretization [27]. The second order upwind differencing scheme was used in the spatial-discretization of the momentum, species, and energy equations [27]. Pressure discretization used the PRESTO! algorithm [27], with the no-slip boundary condition assumption over all wall surfaces.

### 2.3. Laminar-Flow MFC Experiments

**Figure 2:**
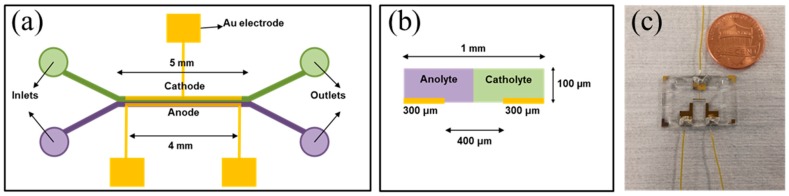
(**a**) A schematic of the laminar-flow microbial fuel cells (MFC) used in the experimental measurements presented in this work. (**b**) The cross-sectional configuration within the mixing region of the laminar-flow MFC. (**c**) An image of the actual laminar-flow MFC is shown in (**a**).

Experiments were conducted to validate the presented simulation results. Polydimethylsiloxane (PDMS, 10:1 mixture, Sylgard 184, Dow Corning, Inc., Midland, MI, USA) microchannels were fabricated using the soft lithography technique. In brief, PDMS was poured onto the silicon master, which had the microchannel pattern. A glass substrate and the PDMS layer were then bonded to each other by oxygen plasma treatment (Harrick Plasma) to seal the channels. The assembled device was then treated with ultra-violet (UV) light for 30 min to sterilization, before bacteria inoculation. Figure 2 shows the schematic of the MFC used in the experiments presented in this paper.

Fluorescent dye (FITC) solution and de-ionized (DI) water were flowed through the inlet channels *y*^+^ and *y*^−^ using a syringe pump for flow visualization. Images were taken using a fluorescent microscope (Zeiss Axio Observer Z1, Carl Zeiss SBE, LLC, Thornwood, NY, USA) equipped with a Hamamatsu CCD camera. An in-house MATLAB program was then used to process the obtained images, to measure the diffusion of the FITC dye into the DI water.

For the laminar flow MFC experiment requiring bacterial growth in the microchannel, *Shewanella oneidensis* MR-1, the most commonly used single-culture microorganisms in the vast majority of MFC experiments reported in the literature, transformed with plasmid p519ngfp (expressing green fluorescent protein) in tryptic soy broth (TSB) culture medium was loaded into the microchannel, and the flow stopped for 6 h to allow initial bacterial attachment. After that, both anolyte (TSB) and catholyte (100 mM ferricyanide working as electron acceptors) were flowed continuously at a flow rate of 20 μL/min using a syringe pump (Chemyx Fusion 200, Chemyx, Stafford, TX, USA). The GFP-*Shewanella oneidensis* MR-1 used in this project was supplied by Dr. M. Y. El Naggar at the University of Southern California. In the experiments, the laminar flow MFC was not degassed, but no gas bubble issue in the channel was observed. Biofilm imaging was performed using the same inverted microscope and CCD camera described above.

Regarding power output measurement, the device was connected to varying load resisters (i.e., external circuit), and the voltage drop across the resisters was measured using a multimeter. Power output was then calculated using Ohm’s law. Nine load resistors in the range of 1 MΩ to 400 MΩ were utilized in this case to obtain the power density curve, where each point in the power density curve represented a measurement for a given load resistor.

## 3. Results and Discussion

### 3.1. Effects of Fluid Velocity

**Figure 3:**
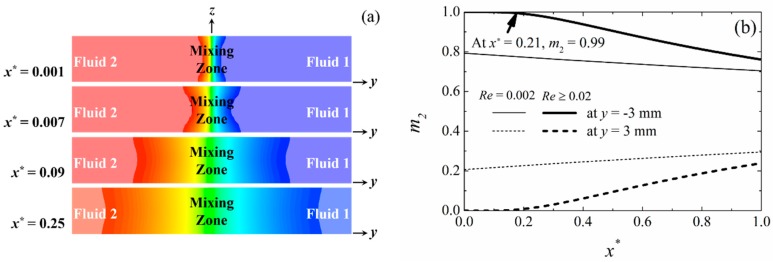
Illustrations of a typical mixing zone in *Channel y*^0^: (**a**) cross-sectional concentration profile, (**b**) mass fraction profile for species 2 along the channel walls (*y* = ±*W*/2) at *z*/*H* = 0.5 and 0.95.

We first briefly looked at the effect of fluid velocity in the range of 1 ≤ *u* ≤ 100 mm/s, corresponding to a Reynolds number of 0.002 ≤ *Re* ≤ 20, on the laminar flow profile. Figure 3a shows the general shape of the mixing region for different normalized downstream location, x^*^, defined as,

(10) $$x^{*}\;=\;\frac{x}{\dot{Q}/D},$$

with a sandglass-shaped cross-section that broadens as it moves away from the center (*z*/*H* = 0.5), the so-called “butterfly effect” [28]. This figure also shows that the length of the non-mixing zone (*λ*) is slightly shorter in the wall region than at the center of the channel (*z*/*H* = 0.5), due to the wider mixing zone close to the channel walls where the fluid velocity was slower. The flow profiles shown in the figure were consistent with the ones previously reported for both T-shape (*θ*_12_ = 180°) and Y-shape (*θ*_12_ = 60°) mixing channels [28,29].

Figure 3b shows the general shape of the mixing zone in *Channel y*^0^ for fluid 2 at *Re* = 0.002 and *Re* ≥ 0.02, which corresponds to flow rates of 2 μL/h and 20 μL/h, respectively. For the case of *Re* = 0.002, streams of fluid were already partially mixed upon entering *Channel y*^0^ (*x* = 0 mm). This is due to the slow fluid velocity that results in a slower convection in the stream-wise direction, compared to the mass diffusion, where fluid 2 and 1 may diffuse “backward” into the inlet *Channels y*^+^ and *y*^−^, respectively. However, as the fluid flow rate increases, convection becomes stronger than the mass diffusion to a point where there is no backward diffusion in the premixing region. Figure 3b summarizes the mass fraction profile in *Channel y*^0^ for fluid 2, for all flows with *Re* ≥ 0.02, where the mass fraction profile in *Channel y*^0^ was found to be similar to the normalized downstream location x^*^. Our result shows that the unmixed region in *Channel y*^0^ extended in the region of 0 ≤ x^*^ ≤ 0.21 regardless of the channel size, indicating a potential optimum operating zone for a given laminar flow fuel cell. Note that this result is valid for any fluid diffusion and flow rates, as long as the flow is laminar.

**Figure 4:**
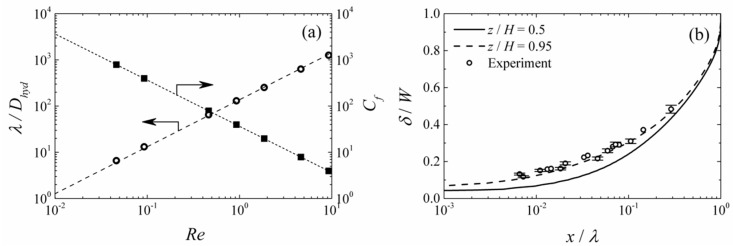
Characteristics of the mixing zone at a different flow rate for the laminar flow MFC system given in Table 2. (**a**) Extent of the unmixed region at z/H=0.5 and wall friction coefficient over the channel wall at *z* = 0. (**b**) Width of mixing zone at *z*/*H* = 0.5 and 0.95. The error bars represent 95% uncertainty level in the experimental measurements.

Figure 4a shows the length of the non-mixing zone *λ* for different fluid velocities (*Re*), normalized by the hydraulic diameter of the channel (*D_Hyd_*). The effects of the Reynolds number on the friction coefficient, *C_f_*, on the microchannel wall surface at z = 0 is shown in Figure 3a as well. It is well established that the wall shear stress and global flow condition on the microchannel wall, affect the growth of biofilm (see Table 1 for the summary) [30,31]. High shear has been shown to limit the growth of biofilm due to cell detachments [20,21,22,23,24,25,32,33]. Some studies showed that erosion of the biofilm layer occurs when the wall shear is higher than 2 to 3 Pa. Thus, the simulation result presented here provided a quick way of understanding the degree of shear stress that will be applied to the biofilm in a particular system geometry and operating condition of choice. For example, the system shown in Table 2 with water as the flowing liquid at 0.023 m/s velocity (*Re* = 5), corresponds to *C_f_* = 10 (*τ* = 2.64 Pa).

Figure 4b shows the profile of the mixing zone width *δ* at *z*/*H* = 0.5 and 0.95. The results of this figure show a higher mixing rate in the regions closer to the inlet at *x*/*λ* < 0.2. Additionally, there existed a small region with a larger mixing width close to the channel wall (*z*/*H* = 0.95). The mixing zone width increased at a faster rate near the wall (*z*/*H* = 0.95) than in the center of the channel. Figure 4b also shows that the growth of the mixing width follows a different scaling law in the interior region, *δ*(*x*) ~ *x*^1/2^, compared to that in the near wall regions, *δ*(*x*) ~ *x*^1/3^, similar to the reported trends in the literature [14]. In summary, the mixing zone width at *z*/*H* = 0.5, was always smaller than that at *z*/*H* = 0.95, as shown in Figure 4b.

Experimental results were also compared to these simulation results (Figure 4b). Here the flow rate was changed from 15 μL/h to 6000 μL/h using a channel with *W*/*H* = 10, and the obtained mixing zone width was measured. The graph shows a very good agreement between the experimental data and the predicted trends for near wall patterns. As the experimental images were taken from the bottom of the PDMS microchannel, the mixing zone width obtained from the image represented the larger mixing width in the channel, i.e., at *z*/*H* = 0.95.

### 3.2. Effects of Angle θ_12_ between the Inlet Channels

**Figure 5:**
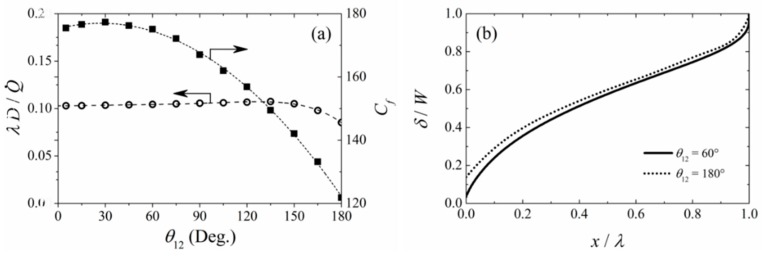
The mixing characteristics for fluid Reynolds number 0.2 in the microchannel system with different inlet angles between *Channel y*^+^ and *Channel y*^−^; (**a**) Normalized length of the unmixing zone and wall friction coefficient against the angle *θ*_12_, (**b**) width of mixing zone at *z/H* = 0.5.

In the previous section, effects of flow rate on the laminar flow profile and shear stress were considered for inlet channel angles (*θ*_12_) of 60°, the so-called Y-shape inlet channels. In this section, the effect of the angle between the two inlet channels is presented for the reference channel dimension in Table 2 and *Re* = 2. Figure 5a shows the length of the unmixed region λ, normalized by the characteristic length scale of *D_j_*/$\dot{Q}$, where $\dot{Q}$ is the volumetric flow rate in the main channel. This figure shows that the length of the non-mixing region is relatively constant for channel angles of less than 135°, and then decreases slightly at larger angles. The normalized length of the non-mixing zone for *θ*_12_ = 180° was 0.085, 18% less than the corresponding value for *θ*_12_ = 60°. This was because the two fluids became partially mixed before they entered the Channel **y**^0^ when *θ*_12_ ≥ 135°, hence the length of the non-mixing zone in the Channel *y*^0^ was reduced with large *θ*_12_. This figure also shows the variation in friction coefficient on Channel *y*^0^ surface at *z* = 0 as a function of *θ*_12_, where the friction coefficient is maximum at *θ*_12_ = 30°, beyond which it starts to drop. Figure 5b compares the profiles of the mixing zone width in the case of the Y-shape inlet channel (*θ*_12_ = 60°) with that of the T-shape inlet channel (*θ*_12_ = 180°). Although the mixing zone for the T-shaped system was wider than that of the Y-shaped system at any given x-direction locations, the difference was relatively small. It is important to note that this result is valid for any fluid at any flow rates, as long as the flow remains laminar.

### 3.3. Effects of Channel Aspect Ratios

**Figure 6:**
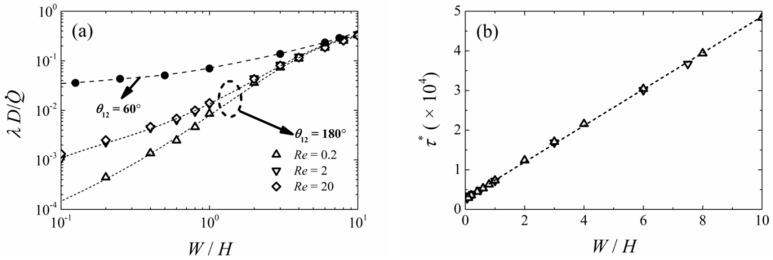
Comparison of the mixing characteristics of a Y-shaped and a T-shaped microchannel system; (**a**) Normalized unmixed region at *z*/*H* = 0.5, as a function of the channel aspect ratio for *θ*_12_ = 60°; (**b**) Normalized wall shear stress against different channel aspect ratio.

Figure 6a shows the variation in *λ* against the channel aspect ratio (*W*/*H*) for inlet channel angles of *θ*_12_ = 60° and 180°, and for flows with Reynolds numbers between 0.2 and 20. The length of the non-mixing region reduced in an asymptotic manner, as the channel aspect ratio decreased for all the Y-shape systems. For the Y-shape inlet channel system, the normalized *λ* was independent of the Reynolds number for all channel aspect ratios. However, the normalized *λ* depended on the Reynolds number for small channel aspect ratios for the T-shape inlet system. On the other hand, the length of the non-mixing zone decreased in a monotonic manner for the Y-shape inlet channels independent of *Re*, whereas a decreasing non-mixing length was observed for the T-shape inlet channel system at *Re* < 2. The difference was because the pre-mixing between the two incoming flows was significantly enhanced for flows with a small Reynolds number in multi-channel systems having a small width to height ratio. Figure 6b shows the normalized wall shear stress on the surface at *x* > 0, −*W*/2 < *y* < *W*/2, and *z* = 0, for both Y- and T-shape inlet channels against channel aspect ratio in the case of *Re* = 2. This figure shows that the normalized wall shear stress was linearly proportional to the channel aspect ratio for laminar flow in any rectangular channels, when presented in terms of τ*.

### 3.4. Asymmetric Channel Cross-Section Due to Biofilm Growth

**Figure 7:**
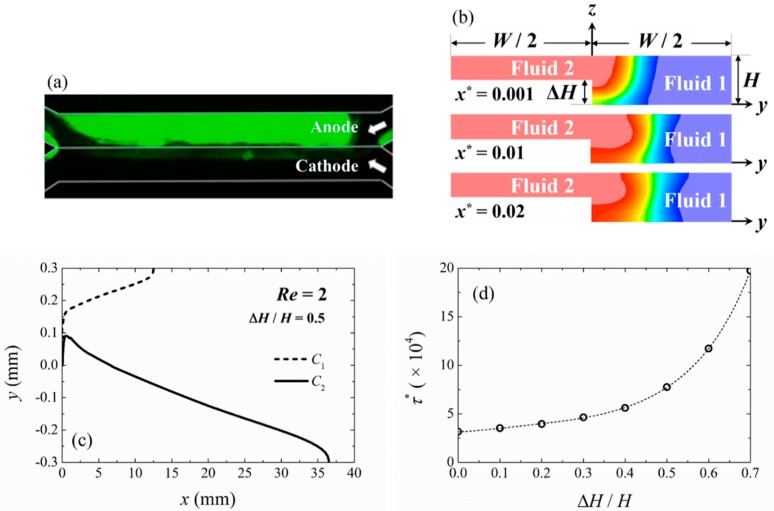
(**a**) Demonstration of biofilm growth inside the laminar flow MFC at a flow rate of 20 μL/min. GFP-*Shewanella oneidensis* MR-1 was flown into the microchannel to form biofilm and observed under a fluorescent microscope after 70 h of growth. (**b**) Concentration profile for fluid 2 at various downstream locations in a microchannel with asymmetric cross-section, where *W*/*H* = 6, Δ*H*/*H* = 0.5 and *Re* = 2. Blue and red colors indicate fluid 1 and 2, respectively. (**c**) The mixing zone profile at *z*/*H* = 0.75 for a channel system with *θ*_12_ = 60°; (**d**) Shear stress variation on the channel wall at *z* = Δ*H* for channels with a different asymmetric cross-section.

**Figure 8:**
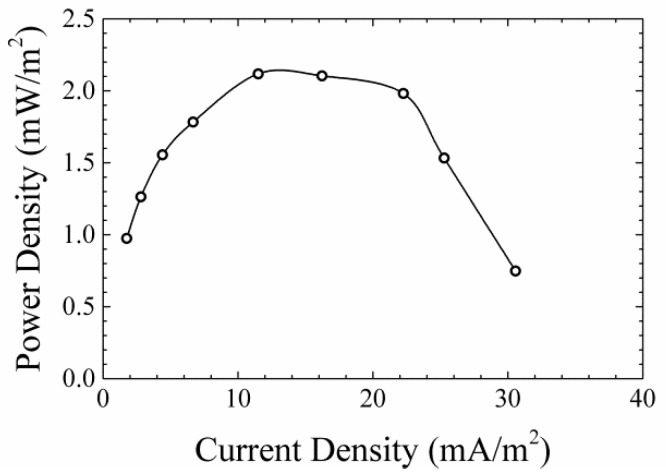
An example power density curve of a laminar-flow MFC device.

When biofilm grows on the anode in a laminar flow MFC system, it effectively affects the channel geometry. Figure 7a shows an example of the growth of the microbial biofilm inside a laminar flow MFC system, which is mostly located on the anode side of the channel. This biofilm grew very thick (visible from the fluorescent intensity of the GFP-expressing microbes), and extended slightly to the cathode side of the channel in about 70 h of growth. It can also be seen that the biofilm growth was irregular and non-uniform. This change in the effective channel geometry due to the biofilm growth was asymmetric, since microbes will mostly grow on the anode side, causing part of the channel height to be reduced and become uneven. This change alters the flow profile inside the channel over time, which affects the mixing dynamics in the laminar flow MFC system, which itself will be affected by the degree of biofilm growth itself on the anode. Figure 8 shows an example of power output from the laminar-flow MFC device, where robust power output from the device can be observed initially. However, after operating the device for more than 70–90 h, we observed no power output due to significant disruption of the laminar flow profile. Here we focused our attention on how this biofilm growth could affect the laminar flow, mixing, and shear stress profiles in the laminar flow MFC system.

A simplified model was developed to study the effects of the biofilm growth by separating the effects of biofilm thickness, and its surface roughness. Here, we first looked at the effect coming from biofilm thickness. The growth of biofilm on the anode side was simulated by assuming a single-step growth on the anode, with a height difference in the range of 0 ≤ Δ*H*/*H* ≤ 0.7 (Figure 7b). Although the actual biofilm within the MFC over time will be non-uniform, the single-step representation correctly describes the physics of flow for a newly inoculated MFC as the initial growth of the biofilm is confined to the anode side due to the initially uniform and undisturbed fluid flow. Since the laminar flow MFC system functions most consistently when it is newly inoculated and only a relatively thin biofilm is formed, the model presented here was deemed sufficient for guiding the design and operation of a laminar flow MFC system. A more sophisticated model, such as a dynamic model simulating the growth of biofilm over time, can be developed in the future based on the current model and experimental data, to provide a more detailed examination of the continuous performance of a laminar flow MFC system over time.

As the biofilm grows, first, the change of geometry into an asymmetric shape will cause the velocity field inside the channel to become asymmetric, where the average velocity of fluid 2 will become higher than that of fluid 1 due to the smaller cross-section. Consequently, in this region, a traverse velocity field (along +*y*-direction) across the channel exists, which pushes fluid 2 into fluid 1. Therefore, mixing in this region is no longer diffusion-limited along the +*y*-direction. Finally, the mixing zone no longer has a regular sandglass-shaped cross-section as shown earlier. Figure 7c shows an example of the mixing zone and the concentration profiles for fluid 2, for an asymmetric channel with Δ*H*/*H* = 0.5. In this case, the length of the non-mixing region *λ*_2_ is about 36 mm for fluid 2, compared to the length of the non-mixing zone *λ*_1_ for fluid 1 of about 12.5 mm, showing strong asymmetry.

Figure 7d shows the normalized wall shear stress on the channel wall at *z* = Δ*H* inside the *Channel y*^0^, for different biofilm thicknesses (Δ*H*). Maximum shear stress always occurs at the anode side (fluid 2 where *y* < 0) of the *Channel y*^0^ due to the higher flow velocity resulting from a smaller cross-sectional area. When Δ*H*/*H* is less than around 0.3 to 0.4, there is a relatively minor change in the shear stress. However, the maximum shear stress increases exponentially beyond that. Biofilms in MFCs typically grow at a speed of 15 to 20 μm/day [34,35]. Hence, this result clearly shows that the growing biofilm will lead to a significant increase in shear stress on the anode biofilm over time.

### 3.5. Effect of Surface Roughness

**Figure 9:**
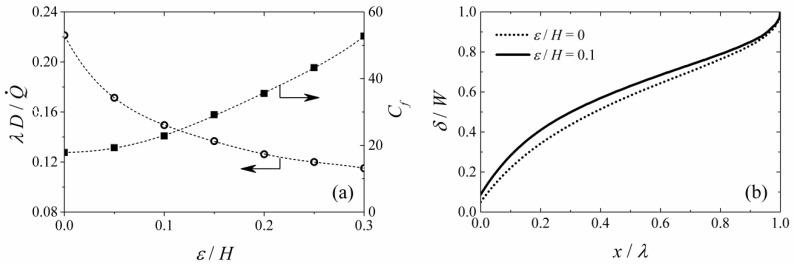
Effects of wall roughness on flow characteristics: (**a**) Length of the non-mixing zone and friction coefficient against normalized wall roughness at *z*/*H* = 0.5 for channel dimension *W*/*H* = 6 and fluid velocity of *Re* = 2. (**b**) Mixing region profile at *z*/*H* = 0.05 for *ε*/*H* = 0.1.

As seen in Figure 7a, the growth of microorganisms is not uniform throughout the channel surfaces. In addition, the channel surface becomes rough over the course of the MFC operation. Surface roughness is known to increase the friction factor and stimulate a turbulence-like fluid motion in the nearby region, which could in turn enhance the mixing process between the incoming fluid streams. Figure 9a shows the normalized length of the non-mixing zone against the normalized wall roughness, within the range 0 ≤ *ε*/*H* ≤ 0.3 (*ε* = 0 representing smooth wall). This figure showed that the wall roughness enhanced mixing between the two fluids because the laminar flow structure was perturbed by the surface roughness as the flow passed over, which created a turbulence-like effect in the close-by region, disturbing the laminar flow within the fluid layer next to the bottom wall. Figure 9b shows the mixing zone profile at *z*/*H* = 0.05, for a flow of *Re* = 2 in a smooth channel (*ε* = 0) and a rough channel with normalized surface roughness (*ε*/*H*) of 0.1. The non-uniformed fluid structure assisted the diffusion processes in a similar manner to turbulence. Moreover, Figure 9a illustrates that surface roughness can significantly increase the induced shear stress, especially when the roughness is more than 10% of the channel height. As indicated before, this is an important factor that can affect the attachment of the microorganisms on the anode.

### 3.6. Effect of Biofluids–Water Diffusion Coefficient

**Figure 10:**
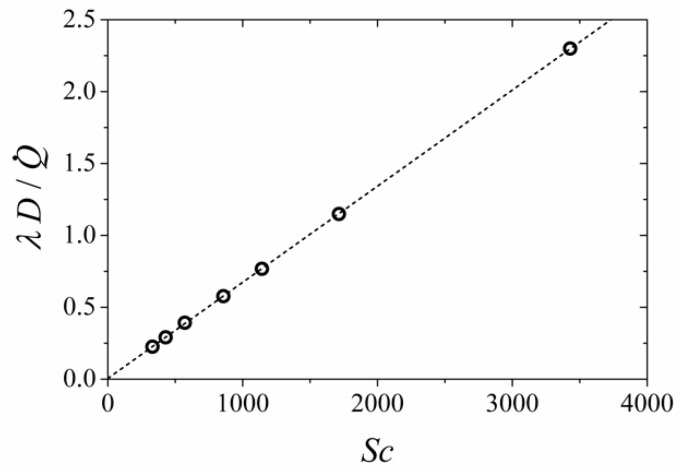
Normalized length of the non-mixing zone for different assumed values of the diffusion coefficient of biofluids.

The mixing process between two parallel laminar flow streams, such as those observed in a laminar flow MFC system, is mainly diffusion-limited where mass diffusivity is the most important parameters. However, diffusivity data of different binary material-pairs are often not readily available, which is especially true for liquids used in most biological applications. Most studies have used water properties in such simulations, with an adjusted value for mass diffusion coefficient. Frequently, in computational simulations, an approximation to the diffusivity is applied in the case when measured data of the diffusivity is unavailable. Typically, the assumed mass diffusivity is in the order of 10^−10^ to 10^−9^ m^2^/ [26], which corresponds to a Schmidt number with an order of magnitude between 100 and 10,000. Figure 10 shows the predicted normalized length of the non-mixing zone, computed when the mass diffusivity, in terms of Schmidt number (*Sc*), is varied between 200 ≤ *Sc* ≤ 3600, with *Re* = 2. The tabulated Schmidt number for water at room temperature was 330, and the corresponding normalized length of the non-mixing zone was 0.24. Most biofluid simulations used an assumed value of 850 to 1700 for the Schmidt number. The assumed values corresponded to a normalized length of the non-mixing zone of between 0.58 and 0.78, much higher than the value predicted using the tabulated value for water. Indeed, the effect of the Schmidt number on the normalized length of the non-mixing zone was linear, and thus this aspect must be further considered to accurately predict the flow behavior in a laminar-flow MFC.

## 4. Discussion and Conclusions

This study presents a computational analysis of the effects of several operating parameters on the fluid flow, mass transfer, and shear stress in a microfluidic laminar flow MFC. The parameters analyzed here were: Effects of fluid velocity in terms of Reynolds numbers, the angle *θ*_12_ between the inlet channels, the ratio of channel width to channel height, channels with asymmetric cross-section due to biofilm growth on anodes, wall roughness due to biofilm growth, and mass diffusion coefficient. The length of the non-mixing zone increased linearly, whilst the friction coefficient decreased linearly with the fluid velocity. Furthermore, the mixing zone width was wider in the close-wall than the interior region of the channel. The angle between *Channel y*^+^ and *Channel y*^−^ had minimal effects on flow characteristics for *θ*_12_ < 135°, and slight effects on systems with *θ*_12_ ≥ 135°. The normalized length of the non-mixing zone shortened in an asymptotic manner for a Y-shape inlet system as the channel aspect ratio reduced, independent of the Reynolds number. On the other hand, the length of the non-mixing zone decreased monotonically with the ratio of channel width to channel height, and it depended on the Reynolds number at small channel aspect ratios (in the case of T-shape inlet channels). For channels with an asymmetric biofilm growth on the anode over time, the normalized shear stress increased exponentially with the growth of the biofilm. The non-mixing region was asymmetric, with a larger length of the non-mixing zone on the side with narrow channel height. In addition, surface roughness can significantly enhance the mixing process. Finally, changing the Schmidt number may have a strong effect on the length of the non-mixing zone, especially for large the Schmidt number, which is typically used in biofluids (corresponding to small mass diffusion coefficient). The presented analysis was shown in dimensionless number, which provided a general design guideline for laminar flow MFCs. This design guideline applies to any channel dimension and any fluid flow parameters, for any condition commonly used in “laminar flow” MFC experiments. Thus, this result can significantly improve the work of researchers who are designing laminar-flow MFCs, compared to the typically utilized trial-and-error based microfluidic device development. In addition, the result also provides guidelines on what flow condition to select, depending on the channel geometry and dimension. The most important parameters to be considered are the fluid velocity, channel cross-section aspect ratio, the inlet channel angle (*θ*_12_), and the diffusivity of the fluid. The first three parameters were shown to have a direct effect on the characteristics of the mixing zone, and fluid diffusivity was shown to directly affect the diffusion behavior between the two fluid streams.

The results presented in this study also provided an analysis of how biofilm growth over time can affect the overall flow profile in a laminar flow MFC. The experimental result that showed how growth in biofilm influences the laminar flow profile was also an important discovery, as disruption in laminar flow profile quickly makes the laminar-flow MFC device incapable of generating any bioelectricity. Thus, this information can help researchers identify what degree of biofilm thickness will disrupt the MFC device, and if needed, create mitigation strategies, such as artificial reduction of biofilm through high-shear-stress application.

Taken together, the presented results can be utilized for both design and operation of laminar-flow MFCs, as well as to better interpret data stemming from such experiments, and thus has broad utility for the MFC community.

## Figures and Tables

**Table 1:** Selected literature that shows the effect of shear stress on the microbial biofilm.

Shear Stress	Reynolds Number	Microbes	Effect
0.005 to 0.02 Pa (13 ≤ *C_f_* ≤ 40)	0.02 ≤ *Re* ≤ 0.07	*P. aeruginosa*	Influence on biofilm biomass [20]
0.08 to 0.2 Pa (24 ≤ *C_f_* ≤ 60)	0.4 ≤ *Re* ≤ 1	Mixture *^A^*	Influence on power output and bacterial composition [21]
0.65 to 0.9 Pa (2 ≤ *C_f_* ≤ 9)	0.55 ≤ *Re* ≤ 1.16	Wastewater	Influence on formed biofilm thickness [22]
0 to 10 Pa (0 ≤ *C_f_* ≤ 4)	1 ≤ *Re* ≤ 2	Wastewater	Influence on biofilm residue after erosion test [23]
0.1 to 13 Pa (0.05 ≤ *C_f_* ≤ 6)	1 ≤ *Re* ≤ 2	Wastewater	Influence on biofilm formation and detachment [24]
0.02 to 0.17 Pa (0.01 ≤ *C_f_* ≤ 0.02)	1000 ≤ *Re* ≤ 4000 *^B^*	*B. cereus*	Influence on biofilm formation and detachment [25]

Notes: *^A^* A mixture of an anaerobic sludge, a soil sample, and an effluent sample from an MFC fed with acetate. *^B^* Defined based on rotational speed.

**Table 2:** Reference case dimensions of the three channels shown in Figure 1 with *θ*_12_ = 60°.

Component	*W* [mm]	*H* [mm]
*Channel y* ^+^	0.5	0.1
*Channel y* ^−^	0.5	0.1
*Channel y* ^0^	1.0	0.1
